# Multipose Face Recognition-Based Combined Adaptive Deep Learning Vector Quantization

**DOI:** 10.1155/2020/8821868

**Published:** 2020-09-24

**Authors:** Shahenda Sarhan, Aida A. Nasr, Mahmoud Y. Shams

**Affiliations:** ^1^Faculty of Computing and Information Technology, King Abdulaziz University, Jeddah, Saudi Arabia; ^2^Faculty of Computers and Information Sciences, Mansoura University, Mansoura, Egypt; ^3^Faculty of Artificial Intelligence, Kafrelsheikh University, Kafr El-Sheikh, Egypt

## Abstract

Multipose face recognition system is one of the recent challenges faced by the researchers interested in security applications. Different researches have been introduced discussing the accuracy improvement of multipose face recognition through enhancing the face detector as Viola-Jones, Real Adaboost, and Cascade Object Detector while others concentrated on the recognition systems as support vector machine and deep convolution neural networks. In this paper, a combined adaptive deep learning vector quantization (CADLVQ) classifier is proposed. The proposed classifier has boosted the weakness of the adaptive deep learning vector quantization classifiers through using the majority voting algorithm with the speeded up robust feature extractor. Experimental results indicate that, the proposed classifier provided promising results in terms of sensitivity, specificity, precision, and accuracy compared to recent approaches in deep learning, statistical, and classical neural networks. Finally, the comparison is empirically performed using confusion matrix to ensure the reliability and robustness of the proposed system compared to the state-of art.

## 1. Introduction

Recently, face recognition has become one of the most discussed topics in the computer vision field, although it is not new but with the revolution in electronic devices as mobile phones researchers have realized that it can be a solution to different issues. Many studies have claimed that they achieved a very high recognition rate of facial images approaching the human recognition rate. This may be true in clear frontal facial images, not noisy or occluded [1, 2]. On the other hand, multipose face recognition with various poses and different head angles will be difficult where multiple snapshots at different time instances should be captured. Multipose face recognition has been discussed lately in different studies where some concentrated on face reconstruction from different head poses using 3D Models [3–5]. Others discussed misalignment, varying illumination, and noise [6–9], while the rest concentrated on achieving higher recognition rate using different techniques as support vector machine [10, 11], Local binary patterns [12], and deep neural networks [13–16].

**Figure 1:**
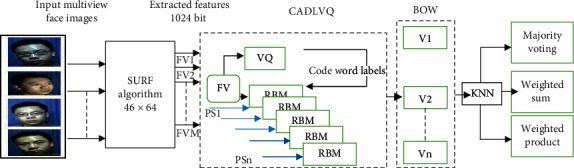
Schematic diagram of the CADLVQ classifier with N parameter sets.

Restricted Boltzmann Machine (RBM) is a generative stochastic unsupervised artificial neural network that can learn a probability distribution over its set of inputs [17–19]. RBM models use stochastic approach instead of deterministic by which the RBM model possesses inherent randomness of a set of parameters values, which leads an ensemble of different outputs. Therefore, in order to make the learning easier, we have restricted the randomness of the parameters values through using RBM as a deep learner and quantizing the vectors based on *k*-means algorithm. In this paper, we introduce a combined adaptive deep learning vector quantization classifier (CADLVQ) with different parameters sets, in which we seek for changing the entire network parameters of adaptive deep learning vector quantization (ADLVQ) to increase the recognition accuracy of multipose face images [19, 20] and to boost the instability and reliability issues of ADLVQ suffering from varied results obtained with each run time. The general schematic diagram of the CADLVQ classifier with different parameters sets is shown in Figure 1.

As clarified in Figure 1, the number of CADLVQ classifiers depends on different parameters sets producing the output classes. And to manage that, we have used the majority voting algorithm to determine the winner class by which the recognition of the system is realized. The contribution of this paper can be summarized as follows:

1. Proposing a combined adaptive deep learning vector quantization classifier to boost the weakness of the ADLVQ classifier
2. Enhancing the stability and reliability of the CADLVQ based multipose face images using different parameters sets
3. Comparing the proposed CADLVQ with the most recent deep learning approaches using matching scores, weighted sum, weighted product, and majority voting
4. Handling colluded face images with different block sizes by predicting the missing features using expectation maximization (EM) algorithm

This paper is organized as follows.  Section 2 covers a brief review of face recognition techniques. In Section 3, the proposed combined adaptive deep learning vector quantization is described in details.  Section 4 introduces simulation and experiments while section 5 comprises results and discussion. Finally Section 6 outlines main conclusions and presents some future work.

## 2. Related Work

### 2.1. Adaptive Deep Learning Approaches

Classical neural networks (NNs) are commonly used to classify the input patterns and objects based on activation function and the weighted summation of the inputs to produce the estimated output values [21]. Online retainable neural network algorithm to retrain the procedure parameter of the network for estimating the optimal selection of the training input and determining the maximum a posteriori as Markov random field presented by Doulamis et al. [22]. To boost weak classifiers in two-classes classification problem, AdaBoost learning algorithm presented by Hastie et. al., [23] has been successfully producing accurate classifiers based on multiclass exponential loss function and forward stage-wise additive modeling.

On the other hand, many researchers investigated using deep learning in face detection as in [24] a detailed survey of deep learning techniques is illustrated which is commonly classified to deep Boltzmann machine (DBM), deep belief neural network (DBN), auto encoder (AE), and Deep convolutional neural networks (DCNN). In [25], the authors studied the effect of sharing of convolutional layers between the region proposal network (RPN) and Fast region-based convolutional neural network (R-CNN) detector module using two datasets. Hao et. al., in [26] used CNN as a base to handle faces of diverse scales through proposing a scale-aware face detector (SAFD) which automatically normalizes face sizes prior to detection. And in [27] Zhu et al. introduced contextual multiscale region-based convolution neural network (CMS-RCNN) to robustly detect human faces and solve occlusions, low resolutions, facial expressions, and illumination variations problems. They tested their proposed system using two datasets containing images collected under various challenging conditions.

Adaptive deep learning fusion paradigm presented by Doulamis and Doulamis [28] can be utilized for tracking objects in stable manner having few labeled data to be trained based on multilayered deep structures and the paradigm dynamically updates the new unlabeled data to adjust the performance of the deep structures.

One of the major problems used in deep learning especially deep convolution neural network is the data compressing for handling the storage of the model. Therefore Gong et. al. [29] utilized k-means clustering approach for vector quantization, which produces a good balance between the model size and the recognition accuracy.

In [30], Doulamis and Voulodimos proposed a fast adaptive learning aslgorithm, named as FAST-MDL, for detecting humans in complex scenes through dynamically updating the parameters of a multilayered deep learning structure. The results indicated that the adaptation of the FAST-MDL was 5 times faster.

For visual domain localization, Angeletti et. al. [31] presented local adaptive (LoAd) deep visual learning by which discriminative information about the training deep models are differentiated and stored in the inner layer of the network. Moreover, an adaptive deep learning model to extract both facial expressions and human actions is presented by Kim and Rhee in [32] to produce human synthetic emotion. Therefore, for autonomous vehicles and surveillance systems, an adaptive deep learning paradigm is presented by Tahboub et. al. [33] to detect the pedestrian of the input video sequences to estimate the detections in the presence of video compression to handle data-rate problem. Doulamis and Doulamis [34] presented an adaptive deep learning architecture based on stacked auto-encoder for fall detection by estimating the trained samples including the humans and background objects and the weights are updated in case of large changes.

Mughees et. al. [35] proposed an algorithm based on spatial updated deep belief network (SDBN) for hyper-spectral image (HSI) classification by which weighted hyper-segmentation for regions is obtained to produce spatial features to be adapted to the actual HSI spatial structures.

### 2.2. Multiview Face Recognition

The improvements of face recognition algorithms are still under research and development. The researchers' efforts are speeded and sustained recently according to the different applications that required more reliable and precise face recognition system.

In [36], both heterogeneous and homogeneous face recognition approaches were investigated, through proposing two methods, a context-aware local binary feature learning (CA-LBFL) and a context-aware local binary multi-scale feature learning (CA-LBMFL). The CA-LBFL is used for exploiting the contextual information of adjacent bits, by constraining the number of shifts from different binary bits, while the CA-LBMFL method is used to jointly learn multiple projection matrices for face representation. Hessian multiset canonical correlations analysis was presented in [37] to tackle this disadvantage of traditional canonical correlations analysis by reducing the extracted multipose features of face images. In [38], a hybrid approach of the kernelized support vector data description (SVDD) and a binary hierarchical decision tree was proposed. The SVDD was used to customize the needed space in large scale face recognition systems through using only the samples that formalize the image set boundaries, while the decision tree was used for enhancing the classification speed. Experiments were conducted using a new database of 285 person prepared by the authors, indicating that the proposed approach has reduced the needed disk space with better classification testing time and without affecting the accuracy.

Many attempts to detect, recognize, identify, and solve different multipose face recognition problems using deep neural networks were presented as in [39], the authors tried to solve the imposter authentication, by using multiple views of a person's face images at different angles achieving an error rate equal to 0.022. While in [19] authentication through proposing a technique based on local gradient pattern with variance (LGPV) and a deep neural network classifier called adaptive deep learning vector quantization was discussed. The LGPV was used to extract the features of the input modalities that are dynamically enrolled in the system, to be classified using deep neural networks (DNN), after quantization using the K-means algorithm based on prior learning vector quantization (LVQ) knowledge.

In [20], we tried to reduce the additive white Gaussian noise effect by proposing a system to detect multipose face images based on Speeded Up Robust Features (SURF), Multi-Layer Perceptron (MLP), and a combined classifier of Learning Vector Quantization (LVQ) and Radial Basis Function (RBF) classifiers. Also in [40], the authors tried to reduce noise through proposing a two stages method, consisting of a data cleaning from noise model and multipose deep convolution neural networks (DCNN) classifier. In [8], convolutional neural networks were also used to improve the region of interest ROI through pooling multiple convolution face images. Two deep learning models, Lightened CNN and VGG-Face, have been used in [41] to solve different face recognition problems as lower and upper face occlusions and misalignment. Experiments results indicated that deep learning can tolerate localizations error of the intraocular distance.

Finally, a couple-agent pose guided generative adversarial network (CAPG-GAN) is proposed in [42] to synthesize face images with different poses, including extreme profile views. Also in [43], extreme pose variations were investigated through proposing a pose-aware convolutional neural network model to handle extreme out-of-plane pose variations of unconstrained face recognition. 3D rendering was used to adaptively model the appearance of face in different poses. Evaluation was conducted using IARPA Janus Benchmark A (IJB-A) and People-In-Photo-Albums (PIPA) datasets, indicating that the proposed model has emulated the accuracy of popular methods that are fine-tuned to the target dataset. In [44], a joint multipose convolutional network to handle landmarks for semi-frontal and profile faces pose variations in-the-wild is proposed. Experiments were conducted using standard static image datasets as IBUG, 300W, COFW, and the latest Menpo Benchmark indicating that the proposed technique achieved superiority over the state-of-the-art. Other papers addressing the face images with different poses could be found in [45–49].

## 3. Combined Adaptive Deep Learning Vector Quantization

As mentioned in section 1, here we introduce a system for multipose face recognition based on combined adaptive deep learning vector quantization. The proposed system is used to handle the problem of fixed parameters investigated in [19]. We are seeking to use variable parameter sets by which enhancement of the whole system will be achieved. During the running process, the results with the fixed parameters are slightly varied. By using variable parameter sets, we ensure the stability of the system results and enhancement of system accuracy.  Algorithm 1 presents the steps of the proposed CADLVQ classifier.

The enrollment stage contains Y multipose facial images with different poses for *M* users entered to the ADLVQ classifiers. Instead of using fixed parameters value, a CADLVQ is performed based on N parameters sets to boost the weakness of ADLVQ classifier. For features extraction of facial images, we used Speeded Up Robust Feature (SURF) extractor, as it is considered one of the most accurate algorithms for facial detection. SURF is a powerful alternative of the Scale Invariant Feature Transform (SIFT) algorithm although it is based on similar properties as SIFT, with a complexity stripped down even further. SURF uses an integer approximation of the determinant of Hessian blob detector, which can be computed with 3 integer operations using a precomputed integral image [50].

ADLVQ, RBM, and vector quantization are used to handle the overfitting problem of the enrolled features while the classification is performed as illustrated in steps from 1 to 7, clarified in Algorithm 1. Five RBMs with five parameter sets are used, where every RBM is tuned by one parameter set in parallel fashion as shown in Figure 1. Each RBM has stacked 20 hidden layers with the Sigmoid function as the activation function. The softmax layer is the final output layer for each ADLVQ classifier.

The initialization step starts with each feature vector of size 1024 from SURF, to be quantized by *K*-means algorithm in order not to consume memory and to prepare the vectors to pretrain using RBM. The codeword for each extracted feature is obtained by the standard vector quantization(VQ) which is used to compress the DNN parameters to improve the system efficiency as it creates a balance between model size and recognition rate, where any missing features or unobserved data undergo feedback to be adapted using vector quantization and DNN classifier.

The obtained codeword from the VQ is then utilized as the class label pretrain for each RBM with cross-entropy as the optimization objective. In order to determine the *k*-clustering of the unsupervised codebook from the extracted SURF features, we started with one cluster and then kept splitting clusters until the points are assigned to each cluster that have a Gaussian distribution.

## 4. Simulation and Experiments

**Figure 2:**
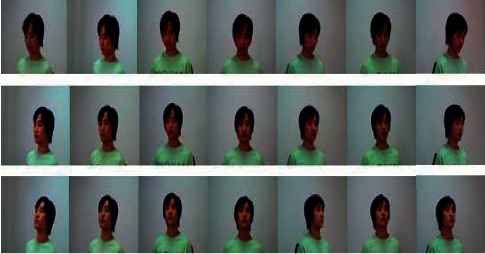
SDUMLA-HMT database [51].

**Figure 3:**
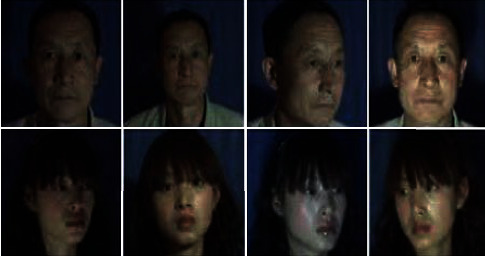
CASIA-FaceV5 database [52].

The CADLVQ was implemented and tested using MATLAB R2019b image processing and computer vision libraries. Experiments were conducted with 3 different views of variant size facial images from SDUMLA-HMT [51] and CASIA-V5 [52], transformed into gray scale with a fixed [64 × 64] image size as input to SURF. SDUMLA-HMT as shown in Figure 2 is a standard database with the images of 106 subjects, 61 males, and 45 females with age between 17 and 31, where each subject is represented by 7 different views of facial images. CASIA-FaceV5 is also a standard database with 500 subjects, represented by 2500 16 bit color BMP facial images as shown in Figure 3, with images of 640 × 480 resolution [53].

## 5. Results and Discussion

As clarified from Table 1, five parameter sets (PS) values for the combined ADLVQ classifier are elected. An example of the voting strength for 10 classes entered the system is illustrated in Table 2.

**Figure 4:**
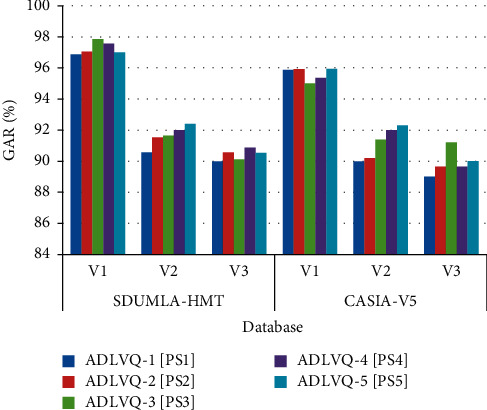
Multipose face recognition based on CADLVQ based on SDUMLA-HMT and CASIA-V5 datasets.

According to the voting strength, the winner class resulting from each classifier is selected. The Genuine Acceptance Rate (GAR) of the tested multipose face images based on CADLVQ classifier is shown in Table 3 from which we can see that five parameter sets were used; V1 represents the frontal view of the face image and V2 and V3 represents different views of the face images. The influence of multipose face images (V1, V2, and V3) on the recognition process with SDUMLA-HMT and CASIA-V5 datasets for CADLVQ with the five parameter sets is shown in Figure 4. It is noticed that an improvement of the GAR (%) is obtained, due to the combined ADLVQ parameter sets variations.

For evaluation the sensitivity, specificity, precision, accuracy, and F1score from the confusion matrix for the proposed system have been calculated based on equations (1)–(5):

(1) $$\begin{array}{c} \text{sensitivity}=\frac{\text{TP}}{\left(\text{TP}+\text{FN}\right)}, \end{array}$$

(2) $$\begin{array}{c} \text{specificity}=\frac{\text{TN}}{\left(\text{TN}+\text{FP}\right)}, \end{array}$$

(3) $$\begin{array}{c} \text{precision}=\frac{\text{TP}}{\left(\text{TP}+\text{FP}\right)}, \end{array}$$

(4) $$\begin{array}{c} \text{accuracy}=\frac{\left(\text{TP}+\text{TN}\right)}{\left(\text{TP}+\text{TN}+\text{FP}+\text{FN}\right)}, \end{array}$$

(5) $$\begin{array}{c} F_{1}\text{score}=\frac{\;2\text{TP}}{\left(2\text{TP}+\text{FP}+\text{FN}\right)}, \end{array}$$

where TP is true positive, TN is true negative, FP is false positive, and FN is false negative.

One of the well-known and reliable benchmarks that summarize the classification performance is the confusion matrix, for an unequal number of observations. Since we have more than two classes, the classification accuracy alone is not sufficient for measuring the system performance. By determining the confusion matrix, a better idea of our CADLVQ classification model will be achieved.

SDUMLA-HMT and CASIA-V5 are used to calculate a confusion matrix with expected outcome values. A comparative evaluation results between the combined learning vector quantization (CLVQ) [54], ADLVQ [19], and CADLVQ are shown in Table 4 in both training and testing phases for SDUMLA-HMT and CASIA-V5. We utilized 318 multipose face images for training and 106 for testing out of SDUMLA-HMT dataset. For CASIA-V5 dataset, we used 2500 multipose face images, 1500 for training and 1000 for testing. The results show the superiority of the proposed system when compared with CLVQ and ADLVQ.

**Figure 5:**
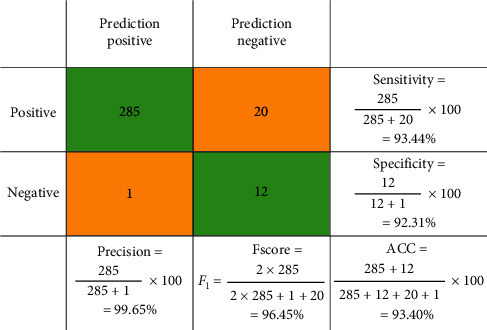
An example for calculating the confusion matrix for the CLVQ based on SDUMLA-HMT dataset in the training phase.

**Figure 6:**
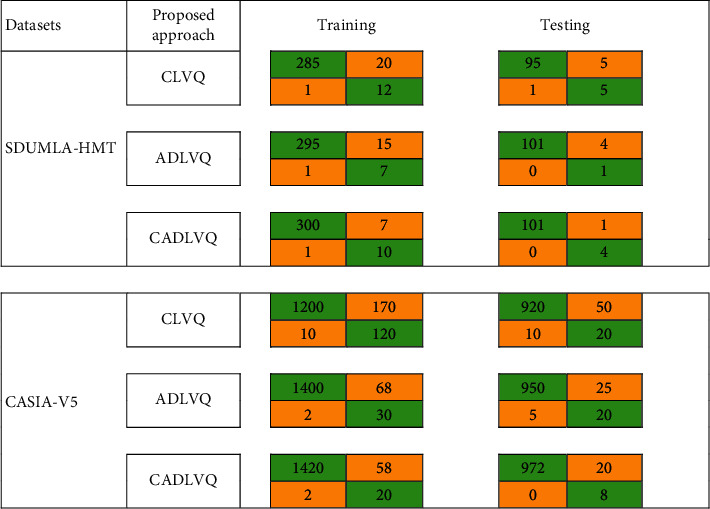
The confusion matrix of CLVQ, ADLVQ, and CADLVQ in training and testing.

An example for calculating the confusion matrix factors is shown in Figure 5. In this example, we used SDUMLA-HMT dataset applied to CLVQ in the training phase by using 318 face images and found that TP = 285, TN = 12, FP = 1, and FN = 20. The training and testing calculations of the confusion matrix of the CLVQ, ADLVQ, and CADLVQ systems based on SDUMLA-HMT and CASIA-V5 datasets are shown in Figure 6.

**Figure 7:**
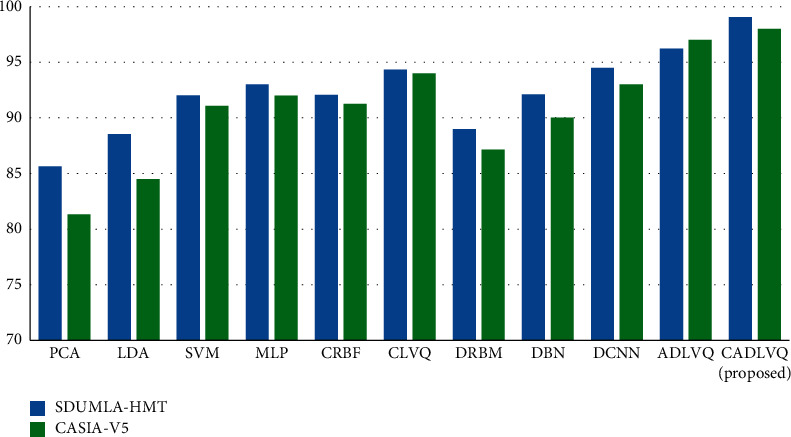
Recognition accuracy.

A comparative evaluation based on the accuracy of CLVQ [54], ADLVQ [19], and the proposed CADLVQ, compared to Support Vector Machine (SVM), Linear Discriminant Analysis (LDA), Principal Component Analysis (PCA), as statistical approach, Multi-Layer Perceptron (MLP), Combined Radial Basis Function (CRBF), as neural network approach, Deep Restricted Boltzmann Machine (DRBM), Deep Belief Neural Nets (DBNN), and Deep Convolutional Neural Network (DCNN) is investigated in Figure 7. The results show that the proposed CADLVQ achieves higher accuracy compared to other approaches.

**Figure 8:**
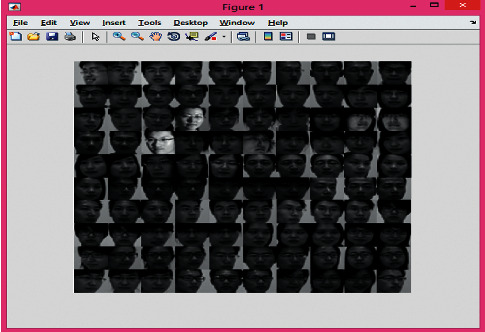
SDUMLA-HMT 100 samples class out of 106 subjects.

**Figure 9:**
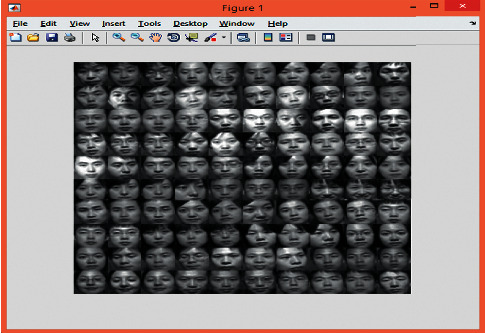
CASIA-V5 100 samples class out of 500 subjects.

Figures 8 and 9, present the resulting 100 samples of multipose facial images obtained respectively from both SDUMLA-HMT and CASIA-V5. While in Table 5, the weighted sum, weight product, and the majority voting results of the CADLVQ are presented, clarifying that a higher accuracy is obtained in case of majority voting than the obtained with the weighted sum, and weighted product, respectively.

**Figure 10:**
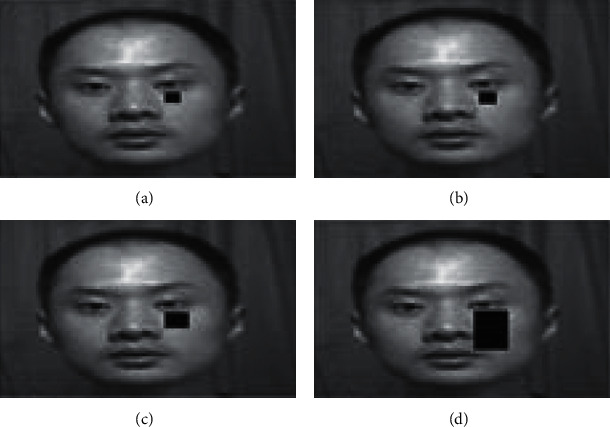
Occluded facial images with different block sizes. (a) 5 ×  5. (b) 6 × 6. (c) 8 × 8. (d) 10 × 40.

A final experiment has been conducted for proving the proposed CADLVQ classifier ability to predict and handle the missing features results from occluded facial images. We empirically tested the proposed system using 40 occluded facial images out of SDUMLA-HMT and CASIA-V5 datasets, respectively. These 40 tested facial images are occluded using different block size images as shown in Figure 10. The recognition accuracy of the 40 occluded facial images with different block sizes is shown in Table 6.

From the above results, we find that restricting the randomness of RBM parameters through using RBM as a deep learner while quantizing vectors based on k-means algorithm has enhanced the recognition accuracy of the RBM. The obtained results were evaluated using large number of facial images with three different views obtained from two different datasets. As is known, the RBM is a generative unsupervised approach that can learn a probability distribution over its set of inputs using stochastic approach instead of deterministic. This stochastic base results in an ensemble of different outputs, so the restriction of RBM parameters randomness results in more efficient training and testing which is clear in enhancing the recognition accuracy of the proposed classifier compared to the state of art using both SDUMLA-HMT and CASIA-V5 datasets as in Figures 6 and 7.

**Figure 11:**
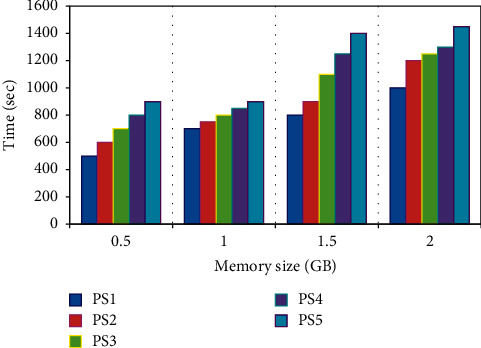
CADLVQ complexity.

The proposed algorithm clarified in Algorithm 1 complexity is calculated as *O*(*M* × *Y* × *N*), based on three factors the number of enrolled users *M*, number of the multipose facial images of each user *Y* and finally the number of the Parameter Sets *N*.  Figure 11 illustrates the variation in consumed memory in GBs versus the time in seconds, with respect to the trained multipose images in SDUMLA-HMT, and CASIA-V5 datasets, respectively. The proposed system accommodated 2 GB in about 30 minutes.

Finally, Table 7 is describing the main advantages/limitations of the proposed method, called CADLVQ and the ADLVQ approach for again clarity of presentation.

As investigated in the table, the main advantage is the obtained accuracy which shows the ability of the proposed CADLVQ to recognize multiview face images. Furthermore, the ability of the proposed to be adapted with different input hyperparameter values as stochastic process to boost weak classifiers in procedure stage. Otherwise, the main limitation is the complexity of the CADLVQ as different training should be executed and we do not think that is not a great problem in the existence of GPU environment and available materials.

## 6. Conclusion and Future Work

In this paper, we present a combined deep learning vector quantization classifier, utilizing different parameter sets to select the winner class using majority voting algorithm to classify and recognize multipose face images. Speeded Up Robust Feature (SURF) was used for extracting the features of facial images, which will be enrolled to the ADLVQ classifier. Experiments were conducted using SDUMLA-HMT and CASIA-V5 standard databases, considering three different views of the face including the frontal view. The proposed classifier performance evaluation was presented as a confusion matrix, in terms of sensitivity, specificity, precision, accuracy, and F1score. Results indicated that the proposed classifier has achieved higher recognition accuracy than ten other classifiers of the state of art. For the future, we will proceed to enhance the proposed classifier performance to be able to handle the spoof attacks problem that may be occurred by fake subjects.

## Figures and Tables

**Algorithm 1:**
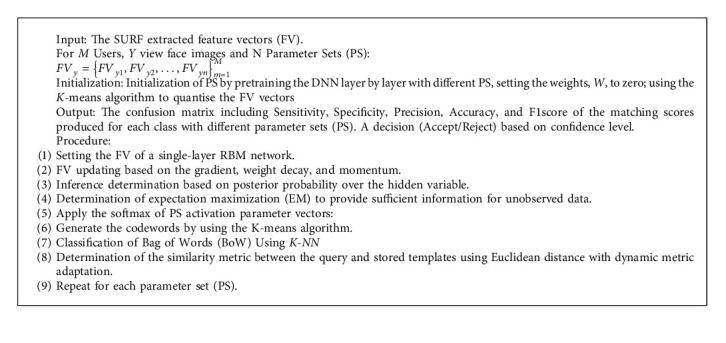
CADLVQ.

**Table 1:** Five parameter sets (PS) for the combined ADLVQ classifier.

	Parameter sets (PS)				
	PS1	PS2	PS3	PS4	PS5
No. of iteration	100	200	300	400	500
Initial momentum	0.2	0.3	0.4	0.5	0.6
Final momentum	0.7	0.8	0.9	0.8	0.9
Weighted cost	0.001	0.002	0.003	0.004	0.005
Learning rate	0.01	0.02	0.03	0.1	0.2

**Table 2:** Voting strength for 10 classes of CADLVQ classifier.

CADLVQ classifier no. [PS]	Voting strength for 10 classes									
	C1	C2	C3	C4	C5	C6	C7	C8	C9	C10
ADLVQ-1[PS1]	1.0	0.9	0.6	0.9	1.0	0.9	0.9	1.0	0.8	0.6
ADLVQ-2[PS2]	0.7	0.6	1.0	1.0	0.7	1.0	0.7	0.6	0.7	0.7
ADLVQ-3[PS3]	0.7	0.8	0.8	1.0	0.8	0.8	0.9	0.9	0.8	0.7
ADLVQ-4[PS4]	0.9	0.6	0.7	0.9	0.6	0.9	0.7	0.7	0.7	0.7
ADLVQ-5[PS5]	1.0	0.8	0.7	0.8	0.6	0.9	0.9	0.6	1.0	0.8

**Table 3:** The GAR (%) of the frontal view (V1), and multipose (V2, V3) for combined ADLVQ classifier.

Multipose face images (V1, V2, V3)	SDUMLA-HMT			CASIA-V5		
	V1	V2	V3	V1	V2	V3
ADLVQ-1[PS1]	96.87	90.56	90.00	95.87	90.00	89.00
ADLVQ-2[PS2]	97.05	91.54	90.56	95.92	90.20	89.64
ADLVQ-3[PS3]	**97.85**	91.65	90.12	95.00	91.38	**91.21**
ADLVQ-4[PS4]	97.56	92.00	**90.87**	95.35	92.00	89.66
ADLVQ-5[PS5]	97.00	**92.40**	90.54	95.95	**92.30**	90.01

V1 (Pose1): frontal view of face image, V2 and V3 (Pose2 and 3) multipose face images.

**Table 4:** The confusion matrix for the proposed CLVQ, ADLVQ, and CADLVQ based on SDUMLA-HMT and CASIA-V5 datasets in the training and testing phases.

DB = SDUMLA-HMT						
	Training (318)			Testing (106)		
	CLVQ	ADLVQ	CADLVQ	CLVQ	ADLVQ	CADLVQ
Sensitivity	93.44	95.16	97.72	95.00	96.19	99.02
Specificity	92.31	87.50	90.91	83.33	100.00	100.00
Precision	99.65	99.66	99.67	98.96	100.00	100.00
Accuracy	93.40	94.97	97.48	94.34	96.23	99.06
F_1_score	96.45	97.36	98.68	96.94	98.06	99.51

DB = CASIA-V5						
	Training (1500)			Testing (1000)		
Sensitivity	87.59	95.37	96.08	94.85	97.44	97.98
Specificity	92.31	93.75	90.91	66.67	80.00	100.00
Precision	99.17	99.86	99.86	98.92	99.48	100.00
Accuracy	88.00	95.33	96.00	94.00	97.00	98.00
F_1_score	93.02	97.56	97.93	96.84	98.45	98.98

**Table 5:** CADLVQ weighted sum, weighted product, and majority voting results.

	SDUMLA-HMT	CASIA-V5
Weighted sum	97.16	95.28
Weighted product	96.25	94.25
Majority voting	99.06	98.00

**Table 6:** Recognition accuracy of the 40 occluded facial images with different block sizes.

Image size format	SDUMLA-HMT	CASIA-V5
5 × 5	93.25	92.65
6 × 6	90.36	90.05
8 × 8	90.01	89.25
10 × 40	85.54	85.08

**Table 7:** Comparison between ADLVQ and CADLVQ.

	ADLVQ	CADLVQ
Hyper-parameters	Fixed	Variable
Adaptation	Adaptation in procedure stage	Adaptation in both procedure stage and hyper-parameter inputs
Complexity	High	Very high
Maximum memory size	1.5 GB	2 GB
Average accuracy	96.62%	98.53%

## Data Availability

The data used in the study are available at http://mla.sdu.edu.cn/sdumla-hmt.html and http://www.idealtest.org/dbDetailForUser.do?id=9.
